# The Role of GITR/GITRL Interaction in Autoimmune Diseases

**DOI:** 10.3389/fimmu.2020.588682

**Published:** 2020-10-09

**Authors:** Jie Tian, Beibei Zhang, Ke Rui, Shengjun Wang

**Affiliations:** ^1^Department of Laboratory Medicine, The Affiliated People’s Hospital, Jiangsu University, Zhenjiang, China; ^2^Department of Immunology, Jiangsu Key Laboratory of Laboratory Medicine, School of Medicine, Jiangsu University, Zhenjiang, China; ^3^Department of Laboratory Medicine, Affiliated Hospital of Jiangsu University, Zhenjiang, China

**Keywords:** glucocorticoid-induced TNFR-related protein, GITRL, T cell, myeloid cells, autoimmune diseases

## Abstract

Glucocorticoid-induced TNFR-related protein (GITR) is a member of the TNFR superfamily which is expressed in various cells, including T cells, natural killer cells and some myeloid cells. GITR is activated by its ligand, GITRL, mainly expressed on antigen presenting cells and endothelial cells. It has been acknowledged that the engagement of GITR can modulate both innate and adaptive immune responses. Accumulated evidence suggests GITR/GITRL interaction is involved in the pathogenesis of tumor, inflammation and autoimmune diseases. In this review, we describe the effects of GITR/GITRL activation on effector T cells, regulatory T cells (Tregs) and myeloid cells; summarize its role and the underlying mechanisms in modulating autoimmune diseases.

## Introduction

Glucocorticoid-induced TNFR related protein (GITR, also known as TNFRSF18) is a member of the TNFR superfamily, which is expressed on regulatory T cells (Tregs) and some activated immune cells, including effector T lymphocytes, nature killer (NK) cells, and neutrophils (1–4). Our previous data have shown that myeloid-derived suppressor cells (MDSCs) could also express GITR (5). GITR is triggered by its ligand, GITRL, which is mainly expressed on B cells, dendritic cells (DCs), macrophages and endothelial cells (6, 7). GITR engagement on effector T cells can generate a positive costimulatory signal and promote T cell activation and proliferation, whereas the activation of GITR on Tregs abrogates their suppressive function (6, 8). In addition, GITR triggering increases resistance to tumors and viral infections, and exacerbates autoimmune diseases and inflammation processes (9). This review mainly focuses on recent studies regarding the GITR/GITRL role in autoimmune diseases.

## Effect of GITR on Effector T Cells

GITR is expressed at low levels on resting CD4^+^ and CD8^+^T cells, and the level of GITR will be up-regulated following T-cell receptor (TCR) activation (8). GITR triggering exerts co-stimulatory effects on conventional T cells by increasing T cell survival, activation and proliferation (3). In fact, GITR stimulated by GITRL or anti-GITR Ab can increase TCR-induced T cell proliferation and cytokine production, and rescues T cells from anti-CD3-induced apoptosis (3, 10). Further investigations indicate a different role of GITR in CD4^+^ and CD8^+^ T cells. It has been acknowledged that the co-stimulatory capability of GITR is weaker and essentially different from that of CD28 (3, 11). Ronchetti et al. indicated that GITR could lower the threshold of CD28 co-stimulation in effector CD8^+^ T cells, and GITR activity on CD8^+^ T cells is considered to be independent of CD28 activation, which is different from CD4^+^ T cells (10, 12). In effector CD4^+^ T cells, the predominated conclusion is triggering GITR on CD4^+^CD25^-^ T cells can induce the survival, activation and proliferation of CD4^+^T cells, and the effect is mainly dependent on TCR stimulation and CD28 co-triggering (13). However, several researches reported that GITR/GITRL interaction could also contribute to immune homeostasis by regulating effector CD4^+^T cells (14, 15). GITR engagement can mediate cell apoptosis. By using a graft versus host disease (GvHD) model, Muriglan et al. showed GITR activation on CD4^+^T cells reduced alloreactive proliferation in mixed lymphocyte reaction (MLR) experiments, and the reduction in expansion was due to the increased apoptosis *via* Fas-FasL pathway (16). Also, It has been found that co-stimulation of GITR showed a potent capacity to produce abundant IL-10, and caused the counter-regulation of increased proliferation responses (17).

**Figure 1:**
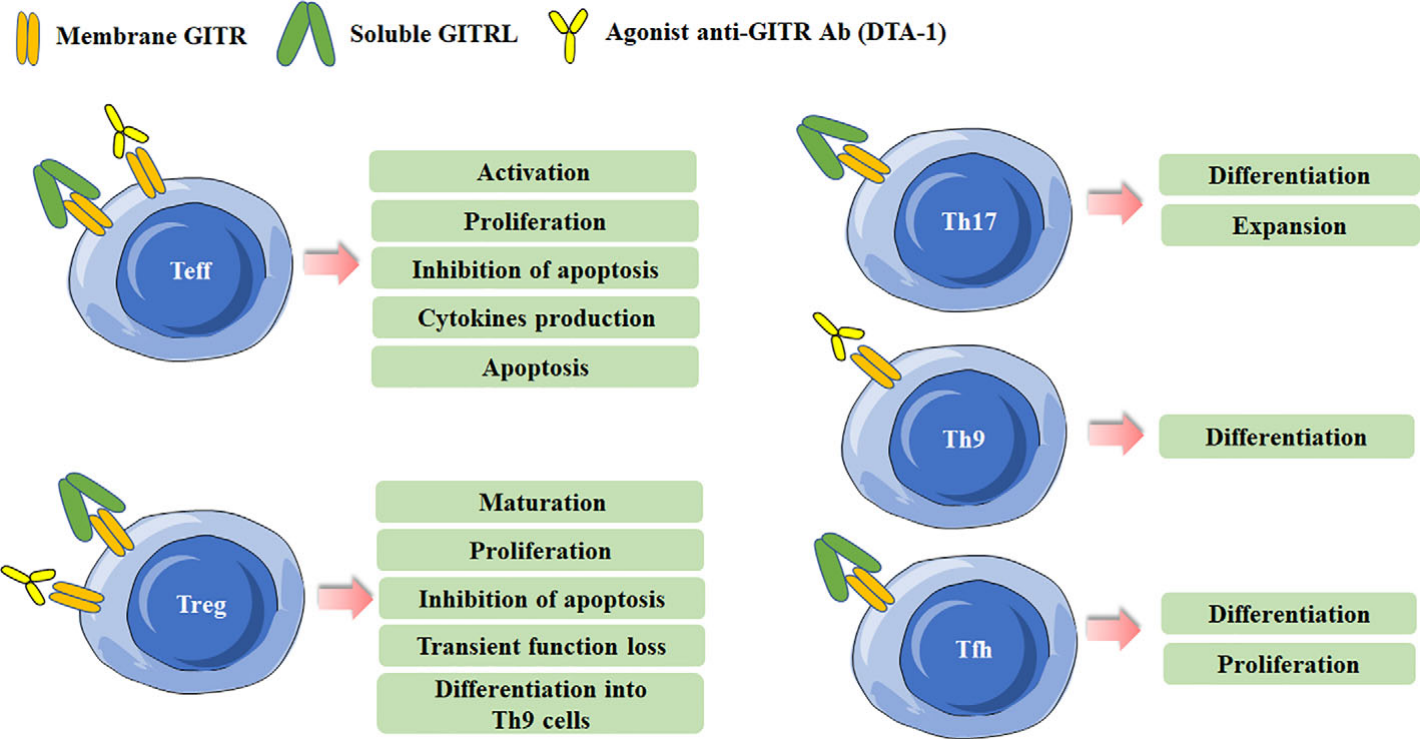
Effects of Glucocorticoid-induced TNFR-related protein (GITR) triggering in effector T cells and Treg cells. Different effects are induced in effector T cells and Treg cells after GITR triggering by GITR ligand (GITRL) or agonist anti-GITR Ab (DTA-1). Triggering GITR can promote the differentiation and proliferation of Th17, Th9, and Tfh cells.

Recent years, the regulation of GITR/GITRL was also investigated in several novel Th (T helper) cell subsets, including IL-17-producing T helper (Th17) cells, T follicular helper (Tfh) cells and IL-9-producing T helper (Th9) cells (18–22). Our previous study has found that recombinant GITRL can promote the differentiation and expansion of Th17 cells (18). Clouthier et al. reported that GITR expression was induced during the maturation phase of germinal center (GC) Tfh cells. Researches have implied that GITR was implicated in the differentiation and function of Tfh cells. GITR deficiency may result in impaired Tfh cell response, considering that Tfh cells in GITR^-/-^ mice showed significant lower frequency and weaker capacity to provide help in antibody production than that in GITR^+/+^ mice (23). Similarly, our previous study also showed that activation of GITR by GITRL could promote the differentiation of Tfh cells (19). Mechanically, GITR triggering initiated downstream canonical nuclear factor-κB (NF-κB) pathway through recruitment of tumor necrosis factor receptor-associated factor 2 (TRAF2) and TRAF5, which was responsible for B-cell lymphoma 6 (Bcl-6) transcription in Tfh cell program (24). Notably, a recent study identified the regulation of GITR signaling on Tfh cells in anti-tumor immune responses. They found that administration of anti-GITR agonistic antibody could induce IL-21-producing Tfh cells, and thus led to enhanced antitumor immune responses (22). Furthermore, GITR co-stimulation could also enhanced the differentiation of Th9 cells in a TRAF6- and NF-κB-dependent manner, and then promoted the tumor-specific cytotoxic T lymphocyte (CTL) responses (20). The effects of GITR triggering in different Th cell subsets are summarized in **Figure 1**.

## Effect of GITR on Treg Cells

GITR is constitutively expressed in Treg cells and is critical for the development and activity of Treg cells (2, 8, 25). The expression of GITR in Tregs can be up-regulated after activation or in tumor microenvironment, and the level of GITR usually positively correlates with the immunosuppressive function of Tregs (26, 27). GITR engagement in Treg cells can have multiple distinct effects. Both *in vitro* and *in vivo* data indicate that GITR activation abrogates the suppressive function of Tregs (2, 8). The initial study reported an agonist anti-GITR Ab inhibits the suppression of Tregs to break the immunological self-tolerance (8). By co-culture of Tregs from GITR^+/+^ mice and CD4^+^ effector cells from GITR^-/-^ mice, Ronchetti et al. further confirmed the GITR activation on Tregs inhibited the suppressive effect of the cells (3). In fact, while GITR triggering inhibits the suppressive activity of Treg, several other studies identified it can induce Treg proliferation and expansion in vitro. The number of Treg cells was lower in GITR^-/-^mice, and the numbers of thymus-derived Treg (tTreg) and peripherally induced Treg (pTreg) cells were higher in GITRL transgenic mice (28–30). Nowakowska and Kissler have found that the number of Treg cells was increased in Ptpn22-deficient mice, but blockade of GITR ligand prevented Treg cell expansion caused by Ptpn22 knockdown, demonstrating the critical role of GITR signaling in regulating the size and composition of Tregs (31). tTreg cell progenitors express high levels of GITR, OX40 and TNFR2, agonists of these TNFRSF members enhance Treg differentiation whereas combined neutralization of their ligands diminishes the development of Treg cells (25). Additionally, GITRL was reported to be dispensable for the development of Treg cells in the thymus. Furthermore, DCs from GITRL^-/-^ mice were less efficient in inducing proliferation of antigen-specific Treg cells in vitro than the cells from WT mice, indicating GITRL on antigen presenting cells is requisite for optimal Treg-mediated regulation of immune responses (32). Taken together, these results indicate that the effects of GITR binding in Tregs depend on the experimental model, the culture condition, the intensity of GITR activation and the pattern in which it is stimulated. The effects of GITR activation on Treg cells are summarized in **Figure 1**.

## GITR/GITRL Interaction in Myeloid Cells

**Figure 2:**
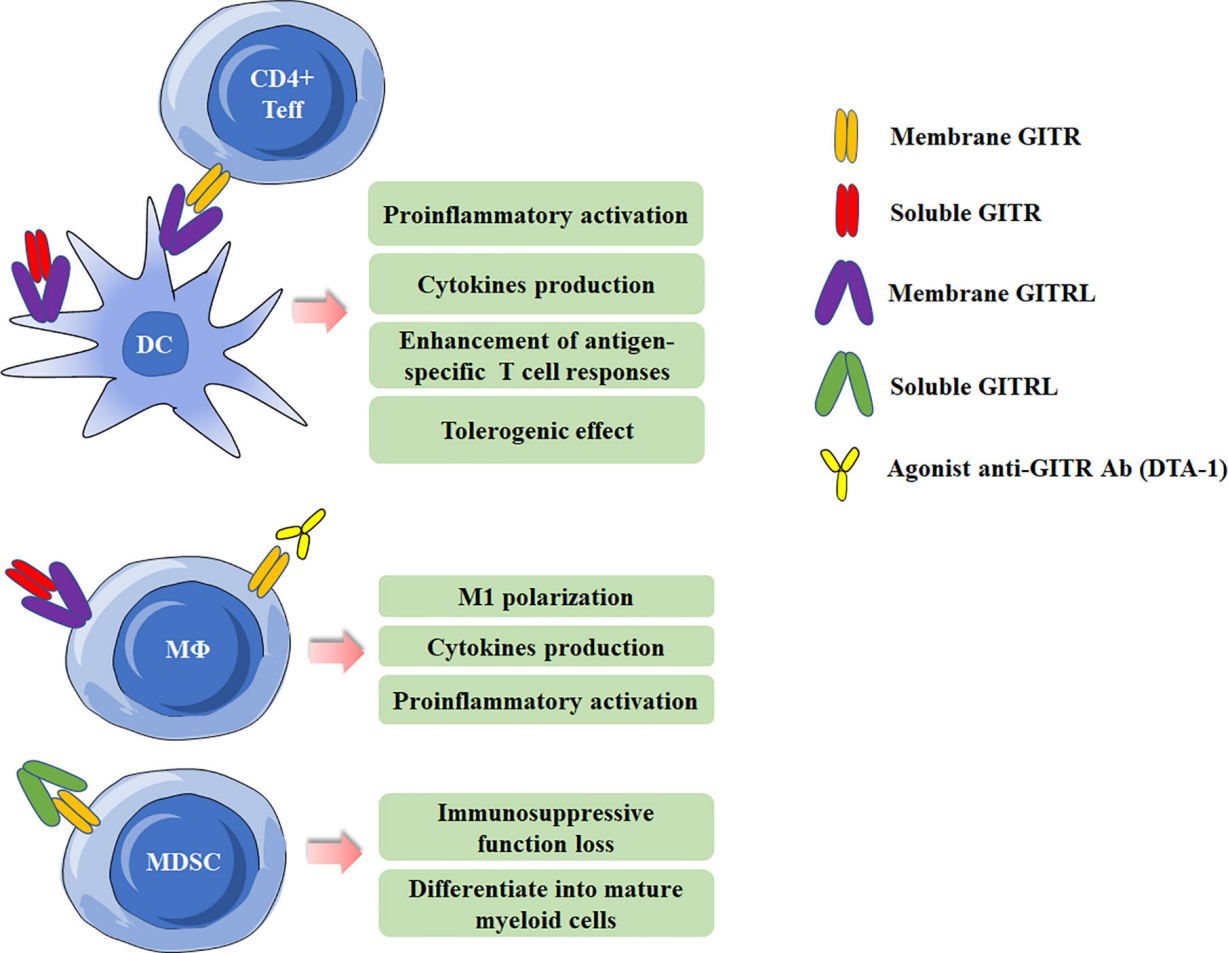
Effects of Glucocorticoid-induced TNFR-related protein (GITR)/GITR ligand (GITRL) activation in myeloid cells. Multiple effects of GITR triggering by agonist anti-GITR Ab (DTA-1) or engagement by soluble/membrane GITR in DCs and macrophages. Activation of GITR by GITRL impairs the suppressive function of MDSCs.

GITR can be detected on various myeloid cells, including monocytes, macrophages, DCs and MDSCs (5, 8, 33–35), and will be at high levels when activated. GITRL can also present on DCs and macrophages. It has been demonstrated that GITRL elicits a tolerogenic effect on plasmacytoid DCs. GITRL initiates the immunoregulatory pathway of tryptophan catabolism by inducing indoleamine-2,3-dioxygenase (IDO) after engagement by soluble GITR (36). In addition, by using the Candida albicans infection model, GITRL triggering downregulated IL-12 production in DCs when they were co-cultured with Treg cells, which acted as a modulator of DC activity (37). However, Ronchetti et al. reported that GITR is important for the activation of DCs and potentiates DC-induced activation of T lymphocytes (38). In macrophages, GITR/GITRL interaction can elicit a proinflammatory effect through both GITRL and GITR activation in macrophages (33). In rheumatoid arthritis (RA) patients, GITR stimulation by anti-GITR mAb increased the production of proinflammatory cytokines/chemokines and matrix metalloproteinase-9 in synovial macrophages (39). Additionally, GITRL triggering by anti-GITRL mAb or soluble GITR can also up-regulated production of proinflammatory and chemoattractant cytokines (33, 40). MDSCs, a population of immature myeloid cells with immunosuppressive function, has been demonstrated be involved in cancer, infectious diseases and autoimmune diseases (41–43). Our previous study has shown MDSCs from experimental Sjögren’s syndrome (ESS) mice could express GITR, and triggering GITR on MDSCs by GITRL significantly reduced the suppressive function of MDSCs on CD4^+^ T-cell proliferation, and the suppressive factors secreted by MDSCs, including arginase and NO, were also down-regulated. The effects of GITR and GITRL triggering in myeloid cells are summarized in **Figure 2**.

## Role of GITR/GITRL Interaction in Autoimmune Diseases

### GITR/GITRL Interaction in RA

RA is a chronic autoimmune disease characterized by persistent synovitis and systemic inflammation, resulting in the cartilage damage and bone erosion (44). In the collagen-induced arthritis (CIA) mouse model, a lower incidence of CIA was induced in GITR^-/-^mice than in GITR^+/+^mice, and less neutrophil infiltration, joint injury, and bone erosion were observed in GITR^-/-^mice. GITR triggering abrogated GITR^+/+^ Treg suppressive effect and co-stimulated GITR^+/+^ CD4^+^CD25^-^ effector T cells, indicating the reduced susceptibility to CIA was due to GITR modulation of effector and Treg cell function (45). Patel et al. demonstrated that activation of GITR by anti-GITR mAb significantly enhanced the production of Th1 and Th2-related cytokines and exacerbated the severity of CIA mice (46).

Of note, the regulation of GITR/GITRL interaction on Th17 and Tfh cells in RA has been explored in recent years. Th17 cells play an essential role in the pathogenesis of autoimmune arthritis (47). Our previous data have shown recombinant GITRL administration could cause an earlier onset of arthritis with markedly increased disease severity and joint damage in CIA mice, and an increasement of Th17 cells were observed in spleen and draining lymph nodes. Further in vitro data revealed that GITRL could efficiently promote naïve CD4^+^T cells differentiate into Th17 cells. All these data identified the function of GITRL in enhancing Th17 differentiation and exacerbating arthritis progression in CIA mice (18). Further studies indicated that GITRL initiated p38 MAPK signal pathway and activated STAT3 signaling, which is responsible for the development of Th17 cells (48). Another novel Th cell subset, Tfh cells, have been reported to be involved in the development of multiple autoimmune diseases, including RA (49). Tfh cells facilitate humoral immunity through supporting GC generation, providing signals crucial for antibody class switching of B cells, generation of high affinity antibodies, and memory formation (50). Ma et al. have found that splenic Tfh cells from CIA mice expressed higher levels of GITR compared with non-Tfh cells, and the activation of GITR significantly enhanced the percentage and number of Tfh cells in vitro and in vivo. Furthermore, blocking the GITR/GITRL by GITR-Fc protein ameliorated the disease severity by suppressing the Tfh cell response (19). Together, all these studies indicate the critical role of GITR/GITRL signaling in CIA mouse model.

In RA patients, the GITRL level was significantly elevated in both serum and synovial fluid (SF). Positive correlations were found between serum GITRL levels and inflammation parameters or autoantibody production. indicating a role of GITRL in the development of RA (51). Additionally, GITR deficiency on RA synovial macrophages has been considered to inhibit the development of autoimmune arthritis via abatement of inflammatory response (39). Accordingly, macrophages function as a proinflammatory agent in the development of autoimmune diseases in a GITR-dependent manner.

### GITR/GITRL Interaction in Primary Sjögren’s Syndrome

Primary Sjögren’s syndrome (pSS) is a systemic autoimmune disease characterized by lymphocytic infiltration of the exocrine glands, such as salivary and lachrymal glands, leading to the loss of secretary function. The primary clinical symptoms are sicca syndrome, including dry eyes and mouth (52, 53). GITRL has been identified to be closely associated with the disease severity in MRL-Fas^lpr^ mice and pSS patients(54, 55). The expression of GITRL in salivary gland duct epithelial cells was evaluated to contribute greatly to the pathogenesis of Sjögren’s syndrome-like autoimmune sialadenitis in MRL-Fas^lpr^ mice (54).The serum GITRL was demonstrated to positively correlate with the degree of lymphocytic infiltration in pSS patients (55). Our recent study further confirmed the role of GITRL in ESS mouse model and pSS patients, and explored the regulation of GITRL on MDSCs in pSS (5). We found that MDSCs gradually lost their suppressive function during the development of ESS, thus leading to progressive inflammation. Further exploration revealed that the increased GITRL in ESS mice could attenuate the suppressive function of MDSCs via activating GITR/GITRL pathway. Moreover, blocking GITR signal in MDSCs significantly restored their immunosuppressive function and ameliorated ESS progression in mice. The similar conclusion was also obtained in pSS patients. All these data identified a critical role of GITRL in modulating the suppressive function of MDSCs, which may facilitate the validation of GITRL as a therapeutic target for the treatment of pSS.

### GITR/GITRL Interaction in Colitis

It has been studied the role of CD4^+^CD25^-^GITR^+^ and CD4^+^CD25^+^GITR^+^ T cells in a well-described CD4^+^CD45RB^hi^ T cell SCID-transferred colitis model. They demonstrated that both CD4^+^CD25^-^GITR^+^ and CD4^+^CD25^+^GITR^+^ T cells, regardless of the CD25 expression, could prevent the development of colitis, indicating CD4^+^CD25^-^GITR^+^ and CD4^+^CD25^+^GITR^+^ T cells can retain the regulatory function (56). Moreover, DTA-1 treatment significantly increased disease severity and death in TNBS-induced colitis (57). Soluble recombinant Fc-GITRL treatment have been reported to exacerbate IBD by inducing the proliferation of pathogenic IFN-γ producing T cells and reducing Treg cells in a mouse model (58). Additionally, GITR deficient mice protected against the colitis by reducing innate immune responses and effector T cell activity. Effector T cells isolated from GITR^-/-^ mice were less effective than T cells isolated from GITR^+/+^ mice to transfer colitis in immunodeficient mice. Blocking the GITR/GITRL signal by soluble GITR prevented the colitis in normal GITR^+/+^ and SCID mice (34). In contrast, Liao et al. evaluated the pathogenesis of colitis by using a CD4^+^T cell transfer model of chronic enterocolitis. The results showed that the expression of GITR on the surface of regulatory and effector CD4^+^T cells was dispensable for progression of the disease, but the presence of GITR on DCs and macrophages was requisite for controlling colitis (59).

### GITR/GITRL Interaction in Other Autoimmune Diseases

Experimental autoimmune thyroiditis (EAT) is a murine model for Hashimoto’s thyroiditis, which is characterized by mononuclear cell infiltration and destruction of the thyroid gland. Administration of anti-GITR mAb in EAT mice inhibits the CD4^+^CD25^+^ T cell mediated tolerance and aggravates the disease EAT, causing the increased autoantibody production and mononuclear infiltration in local tissues. Additionally, our group also found that the upregulated serum GITRL has a positive correlation with the percentage of Th17 cells in Hashimoto’s thyroiditis patients, and the increased GITRL may impair the balance of Th17/Treg, thus contributing to the pathogenesis of Hashimoto’s thyroiditis (60). In an experimental autoimmune encephalomyelitis (EAE) mouse model, It has been observed that anti-GITR Ab could significantly aggravate the disease severity and induce antigen-specific T cell proliferation and cytokine production (61). Also, the role of GITR in systemic lupus erythematosus (SLE) patients was explored. The expression of GITR on Tregs and CD4^+^CD25^-^ responder T lymphocytes (Tresps) were positively correlated with the severity of the disease. Glucocorticoid may achieve its therapeutic effect partly by inducing GITR expression on Tresps rather than Tregs, which initiates the apoptosis of Tresp cells in SLE patients (62).

## Modulation of GITR/GITRL in the Treatment of Autoimmune Diseases

The involvement of GITR/GITRL in both innate and adaptive immunity may account for the inflammatory activation in autoimmune diseases (63). Thus, it is plausible that GITR blockade should be a potential treatment for autoimmune diseases, for inhibiting the activation of autoreactive T lymphocytes and inflammatory cells, and sustaining the immunocompetence of Tregs and MDSCs, which eventually corrects excessive autoimmunity. Although several researches have confirmed the therapeutic effects of GITR-Fc fusion protein or GITR gene knockout in murine autoimmune diseases models, the more explicit impact of GITR-Fc fusion protein on autoimmune microenvironment still needs future study prior to clinical trials (45).

## Conclusion

Researches in recent years have described the roles of GITR/GITRL signaling on various immune cells involved in autoimmune diseases from different perspectives, including Th17 cells, Tfh cells, macrophages and MDSCs. In general, GITR triggering plays a proinflammatory role in the pathological mechanism of autoimmune diseases. Hence, GITR/GITRL system is a potential target for the immunotherapy of autoimmune diseases. Current studies have affirmed the therapeutic effects of GITR-Fc fusion protein in autoimmune diseases murine models, and future researches are expected to clarify the potential mechanism of this agent before application in clinical tests.

## Author Contributions

JT and BZ drafted the manuscript. KR discussed and revised the manuscript. SW designed the study and revised the manuscript. All authors contributed to the article and approved the submitted version.

## Funding

This work was supported by the National Natural Science Foundation of China (Grant Nos. 81971542, 81701612), Natural Science Foundation of Jiangsu (Grant No. BK20170563), Summit of the Six Top Talents Program of Jiangsu Province (Grant No. 2017-YY-006) and Jiangsu Province’s Key Medical Talents Program (Grant No. ZDRCB2016018).

## Conflict of Interest

The authors declare that the research was conducted in the absence of any commercial or financial relationships that could be construed as a potential conflict of interest.

## Abbreviations

Bcl-6, B-cell lymphoma; CIA, collagen-induced arthritis; DCs, dendritic cells; EAE, experimental autoimmune encephalomyelitis; EAT, experimental autoimmune thyroiditis; ESS, experimental SS; GC, germinal center; GITR, glucocorticoid-induced TNFR-related protein; GITRL, GITR ligand; GvHD, graft versus host disease; IDO, indoleamine-2,3-dioxygenase; MDSCs, myeloid-derived suppressor cells; MLR, mixed lymphocyte reaction; NF-κb, nuclear factor-κB; NK, nature killer; pSS, primary Sjögren’s syndrome; pTreg, peripherally induced Treg; RA, rheumatoid arthritis; SF, synovial fluid; SLE, systemic lupus erythematosus; TCR, T-cell receptor; Treg, regulatory T cells; Tfh, T follicular helper; Th, T helper; Th17, IL-17-producing T helper; Th9, IL-9-producing T helper; Tfh, T follicular helper; TRAF2, tumor necrosis factor receptor-associated factor 2; Tresps, responder T lymphocytes; tTreg, thymus-derived Treg.
